# Osteoclast-Mediated Cell Therapy as an Attempt to Treat Elastin Specific Vascular Calcification

**DOI:** 10.3390/molecules26123643

**Published:** 2021-06-15

**Authors:** Chartrisa LaShan Simpson, Jenna A. Mosier, Narendra R. Vyavahare

**Affiliations:** 1 Department of Biological Engineering, Mississippi State University, 130 Creelman Street, Mississippi State, MS 39762, USA; jmosier243@gmail.com; 2 Department of Bioengineering, 301 Rhodes Research Center, Clemson University, Clemson, SC 29634-0905, USA; narenv@clemson.edu

**Keywords:** cell therapy, diabetes mellutis, elastin, microbead, osteoclast, RANK, vascular calcification

## Abstract

Inflammation and stiffness in the arteries is referred to as vascular calcification. This process is a prevalent yet poorly understood consequence of cardiovascular disease and diabetes mellitus, comorbidities with few treatments clinically available. Because this is an active process similar to bone formation, it is hypothesized that osteoclasts (OCs), bone-resorbing cells in the body, could potentially work to reverse existing calcification by resorbing bone material. The receptor activator of nuclear kappa B-ligand (RANKL) is a molecule responsible for triggering a response in monocytes and macrophages that allows them to differentiate into functional OCs. In this study, OC and RANKL delivery were employed to determine whether calcification could be attenuated. OCs were either delivered via direct injection, collagen/alginate microbeads, or collagen gel application, while RANKL was delivered via injection, through either a porcine subdermal model or aortic injury model. While in vitro results yielded a decrease in calcification using OC therapy, in vivo delivery mechanisms did not provide control or regulation to keep cells localized long enough to induce calcification reduction. However, these results do provide context and direction for the future of OC therapy, revealing necessary steps for this treatment to effectively reduce calcification in vivo. The discrepancy between in vivo and in vitro success for OC therapy points to the need for a more stable and time-controlled delivery mechanism that will allow OCs not only to remain at the site of calcification, but also to be regulated so that they are healthy and functioning normally when introduced to diseased tissue.

## 1. Introduction

Vascular calcification is the pathological deposition of the mineral hydroxyapatite in the intimal or medial layers of arteries. Intimal calcification is associated with the inflammation and plaque build-up caused by atherosclerosis. Medial calcification, also known as Mönckeberg’s arteriosclerosis, is most often found in patients with chronic kidney disease and diabetes mellitus, characterized by mineral deposition along the elastin lamellae. In diabetes, arterial beds are damaged due to ruptured plaque deposits [1]. Mineralization is correlated with increased arterial stiffness and a higher risk of mortality from cardiovascular complications [2]. While surgical options such as atherectomy, balloon angioplasty, and stents can be used to prevent the occlusion of arteries caused by the accumulation of plaque, there are no clinically available treatments to prevent or reverse medial calcification.

Over the past decade, it has generally been accepted that vascular calcification is an active cell-mediated process similar to bone formation. The resident vascular smooth muscle cells (VSMCs) in the medial layer undergo a phenotypic switch into osteoblast-like cells, characterized by the loss of smooth muscle contractile proteins (smooth muscle α-actin, smooth muscle myosin heavy chain, SM22-α) and increased expression of bone markers and proteins (Runx2, osteopontin, osteocalcin, alkaline phosphatase) [3,4]. This phenotypic switch can be caused by increased levels of calcium and phosphorus, but other factors such as extracellular matrix (ECM) degradation/remodeling, matrix vesicle formation, apoptotic bodies, and oxidative stresses have also been correlated [3,4,5,6,7,8,9].

In type 2 diabetes mellitus, increased glucose levels lead to increased expression of the runt-related transcription factor (Runx2), also known as the core binding factor alpha-1 (Cbfa-1), and growth factor bone morphogenic protein (BMP-2) [10]. This increase of calcification factors in the serum leads to increased medial inflammation. The medial calcification induced by the high glucose level characteristic of type 2 diabetes is similar to that associated with cardiovascular disease [11,12].

Because of the physiological similarities between vascular calcification and bone formation, we determined that osteoclasts (OCs), bone-resorbing cells that work in concert with osteoblasts during bone remodeling, could be used to treat and reverse medial calcification. In our previous work, we proved that OCs are capable of demineralizing calcified elastin from a subdermal rat model in vitro without any significant damage to the elastin [13]. Co-implantation of allogeneic OCs and elastin subdermally in rats was also able to significantly decrease the extent of calcification without affecting the structure of the elastin implant for up to seven days [13]. While this study showed promise, it did not look at the more clinically relevant effects in vivo. OCs were also not present in the elastin implant after seven days, revealing the need for a cell encapsulation system to deliver and maintain viable OCs for extended periods of time.

Cell encapsulation can be done through macro-platforms (bulk hydrogels, fibers, beads) or micro-platforms (microbeads) using natural or synthetic polymers. Collagen is a natural polymer and the most abundant protein found in mammalian tissue that can self-assemble strands to create hydrogels, making it a popular choice [14]. Our previous research has shown that collagen hydrogel is a suitable vehicle for OC delivery up to seven days [13]. For long-term encapsulation, alginate is a potential candidate as it does not typically degrade in vivo. It is ideal for cell encapsulation because it has the ability to crosslink in mild conditions suitable for cell viability and is biocompatible [15].

Cell encapsulation research has predominantly focused on allowing allogeneic or xenogeneic cells to release therapeutic molecules through a semipermeable membrane without causing a host immune reaction. Considerable research has been completed on the encapsulation and implantation of pancreatic islet cells for the treatment of type 1 diabetes. In pre-clinical research, encapsulated islet cells in alginate beads were able to remain viable and normalize glucose levels after an extended period of time in vivo [16,17]. Recently, cell encapsulation has been used to retain cells at a specific location or to deliver cells. In a study investigating myocardial infarction treatment, Mayfield et al. showed that encapsulated cells survived longer than unencapsulated cells, allowing a longer release of beneficial molecules [18]. Zhou et al. used fast degrading microbeads as a vehicle that could protect the encapsulated cells during injection into a scaffold and then quickly release the cells to promote tissue regeneration [19]. These studies, as well as our previous work, show potential for utilizing cell encapsulation techniques for the delivery and retention of OCs for vascular calcification treatment.

However, delivering OCs is only one part of the potential therapeutic process. To continue, native OCs may also need to be recruited. The receptor activator of nuclear factor-kappa B ligand (RANKL) is a gene hypothesized to be heavily involved in the calcification process in vasculature. In bone, RANKL acts by binding to the RANK receptors on macrophages, causing them to differentiate into OCs [20]. Decreased levels of RANKL have proven effective in the treatment of bone diseases like osteoporosis [21]. Osteoprotegerin (OPG) is a decoy for RANKL that binds to the RANK receptors to prevent osteoclastic differentiation. Therefore, it is believed that the addition of RANKL at the site of calcification in arteries could be successful in activating OC-like cells to resorb mineral deposits.

In this study, the viability and migration of OCs encapsulated in a collagen hydrogel and collagen/alginate beads after implantation was tested. RANKL injections were also used to potentially activate native OC-like cells to work in conjunction with introduced OCs for the purpose of calcification resorption. The study also explored the ability of these implanted OCs to reverse pre-existing calcification and cause a change in gene expression in two different models for vascular calcification, specifically, a rat subdermal implantation model and an abdominal aortic injury model. Results reveal that with modifications, these methods may have the potential to inhibit medial calcification.

## 2. Materials and Methods

### 2.1. Microbead Osteoclast Delivery

To determine the effectiveness of direct OC treatment in calcified regions, OCs were isolated and differentiated with Vitamin D3 with retinoic acid media supplementation as previously described [13]. For elastin calcification studies, porcine aortic elastin was purified and subdermally implanted. The elastin was allowed to calcify for seven days before cell therapy treatment.

#### 2.1.1. Collagen/Alginate Bead Preparation and Testing

Rat skin fibroblasts were fluorescently labeled with CellTrace™ Far Red DDAO-SE (Molecular Probes, Carlsbad, CA, USA) then centrifuged. After the cell pellet was added to the 0.1% *w/v* sodium alginate (Sigma, St. Louis, MO, USA) with 0.1% *w/v* bovine tendon collagen (EPC), the mixture was taken up into a syringe and placed into a syringe pump (Medfusion Injector, Medex Inc., Yellowknife, NT, USA). The solution was then pushed through a 21G needle and dropped into a gently stirring 2% *w/v* calcium chloride (Sigma, St. Louis, MO, USA). Once the mixture was completely dispensed, the beads were allowed to stir for one hour to ensure complete crosslinking and were then washed three times in DI water. The entire process was performed sterilely.

After rinsing, the beads were placed into well plates and cultured for seven days in DMEM with 10% FBS and 1% Ab/Am. On day seven, media was removed and analyzed with MTS (Promega, Madison, WI, USA) Cell Viability assay. After removing beads, a picogreen DNA assay (Invitrogen, Carlsbad, CA, USA) was performed on cells remaining in the culture dish to determine if there was any cell proliferation from the beads. After confirming the success of fibroblast viability, beads could be used for further experimentation.

#### 2.1.2. Osteoclast Labeling and Preparation

OCs were delivered using three methods: direct injection, encapsulation in gel, or encapsulation in beads. For the first method, mature OCs, BMPCs, and rat skin fibroblasts were suspended in sterile PBS (1 × 10^5^ cells/0.5 mL), with the control group using saline injections (0.5 mL/injection). Solutions were sterilely transferred to the surgery suite for injections.

For the second and third methods, mature OCs were labeled using CellTrace™ Far Red DDAO-SE (Molecular Probes, Carlsbad, CA, USA) and were either encapsulated in collagen gel or collagen/alginate beads. Labeled cells to be encapsulated in the gel were added to a collagen solution previously prepared with eight parts PureCol^®^ (Advanced BioMatrix, San Diego, CA, USA), one part 10× Phosphate Buffered Saline (PBS) and one part 0.1 M Sodium Hydroxide (NaOH) in a 50 mL centrifuge tube on ice. The mixture was vortexed vigorously and pH adjusted to 6.5–7.0 with 0.5 M Hydrochloric Acid (HCl). The cell/collagen mixture was taken up into 1 mL syringes (with tips removed for easier implantation) with a cell concentration of 5 × 10^5^ cells/gel and allowed to solidify sterilely at 37 °C for ~30 min. The control groups included collagen only. Once solid, gels with encapsulated DDAO-labeled OCs were sterilely transferred to the surgery suite.

Collagen/alginate beads were prepared as described previously, this time using labeled OCs, and transferred to the surgical suite for implantation.

#### 2.1.3. Treatment Delivery

Animals with implanted calcified elastin were brought to the surgical suite. For injections, animals were held by an assistant while the injections were delivered directly to the implant site. For gel and bead delivery, animals were placed under general anesthesia (2% isoflurane) then two incisions were made on the back above the shoulder blades (left and right side) followed by the formation of subdermal pockets by blunt dissection to expose the previously implanted elastin. Either collagen gels or collagen/alginate beads were placed next to the elastin implant and the site was closed with staples. All animals were humanely euthanized using CO_2_ asphyxiation seven days following treatment. All animal studies were performed according to protocols approved by the Clemson University Animal Research Committee. Animal care was provided by trained veterinarians according to the NIH guidelines for the care and use of laboratory animals.

### 2.2. RANKL Injection

#### RANKL/OPG Injections

Porcine aortic elastin was purified the same as before and implanted subdermally. The elastin was allowed to calcify for seven days. On day seven, injections began for recruitment. RANKL and OPG were delivered into each implant twice daily at doses of 0.4 mg/kg and 0.5 mg/kg, respectively (*n* = 4). Control injections of saline were delivered twice daily at a volume of 0.1 mL/implant (*n* = 4). The animals were humanely euthanized using CO_2_ asphyxiation seven days later on day 14.

### 2.3. Immediate OC-Encapsulated Collagen Gel Application

#### 2.3.1. Abdominal Aortic Injury Surgery

OCs were isolated and encapsulated in collagen gel using the same methods as described previously. Adult male Sprague-Dawley rats (Harlan Laboratories, Indianapolis, IN, USA) weighing ~250 g were placed under general anesthesia (2% isoflurane) then the infrarenal aorta (between the renal artery and iliac bifurcation) was exposed and 0.15 M CaCl_2_-soaked sterile cotton gauze was applied for 15 min to induce calcification. The gauze was then removed and the abdominal cavity was rinsed three times with warm sterile saline. The experimental group (*n* = 5) received the application of the collagen gel with the encapsulated DDAO-labeled OCs in the previously treated aorta while the control group (*n* = 5) received none. The abdominal cavity was closed, followed by the use of subcutaneous sutures and staples. The animals were humanely euthanized on day seven and each injured aorta was retrieved and processed for histological analysis, calcium and desmosine content, and gene expression.

#### 2.3.2. Histology

Following euthanasia, the aortic tissue was embedded in OCT (Sakura Finetek, Torrance, CA, USA) and frozen on dry ice. The frozen tissue was later sectioned and stained for calcium deposits following Dahl’s Alizarin Red procedure. Sectioned and unstained tissue was cover-slipped with DAPI mounting media (Molecular Probes, Carlsbad, CA, USA) to fluorescently image the implanted cells.

### 2.4. Analysis

#### 2.4.1. Calcium and Desmosine Analysis

The capsule was removed from all explanted samples, which were then frozen at −80 °C and lyophilized. The dried samples (~10–15 mg) were hydrolyzed in 6 N HCl in a boiling water bath. The hydrochloric acid was evaporated using a continuous stream of nitrogen gas then reconstituted in 0.01 N HCl. Calcium content was measured using atomic absorption spectrophotometry (Aanalyst 200, Perkin-Elmer, Norwalk, CT, USA). The same hydrolysates were analyzed for desmosine content using radioimmunoassay [19].

#### 2.4.2. RNA Extraction and Gene Analysis

Total RNA was isolated from tissue explants using the RNeasy Fibrous Kit (Qiagen, Valencia, CA, USA). The quality and quantity of RNA were evaluated on an Agilent 2100 Bioanalyzer using the RNA 6000 Nano kit (Agilent Technologies Inc., Foster City, CA, USA). Of the total RNA, 500 ng was then reverse transcribed using RetroScript Kit (Ambion, Austin, TX, USA). The cDNA sample was further amplified using a Rotorgene 3000 thermal cycler (Corbett Research, Mortlake, NSW, Australia) and QuantiTect SYBR Green PCR kit (Qiagen, Valencia, CA, USA), which allows real-time quantity detection of PCR products. Target-specific primers were synthesized by Integrated DNA Technologies Inc. (Coralville, IA, USA). Minus RT and minus cDNA samples were included in each set of samples. Each sample was normalized to the expression of GAPDH as a housekeeping gene and compared to control samples (CaCl_2_ only) using the 2^−ΔΔC^_T_ method.

#### 2.4.3. Statistical Analysis

GraphPad Prism software, version 8.4.3 was used for statistical analysis. Data are reported as means ± SEM. A one-way ANOVA followed by a post hoc Student’s *t*-test were used to determine differences between groups. Data are determined statistically significant when *p* < 0.05.

## 3. Results

### 3.1. Microbead Osteoclast Delivery

**Figure 1:**
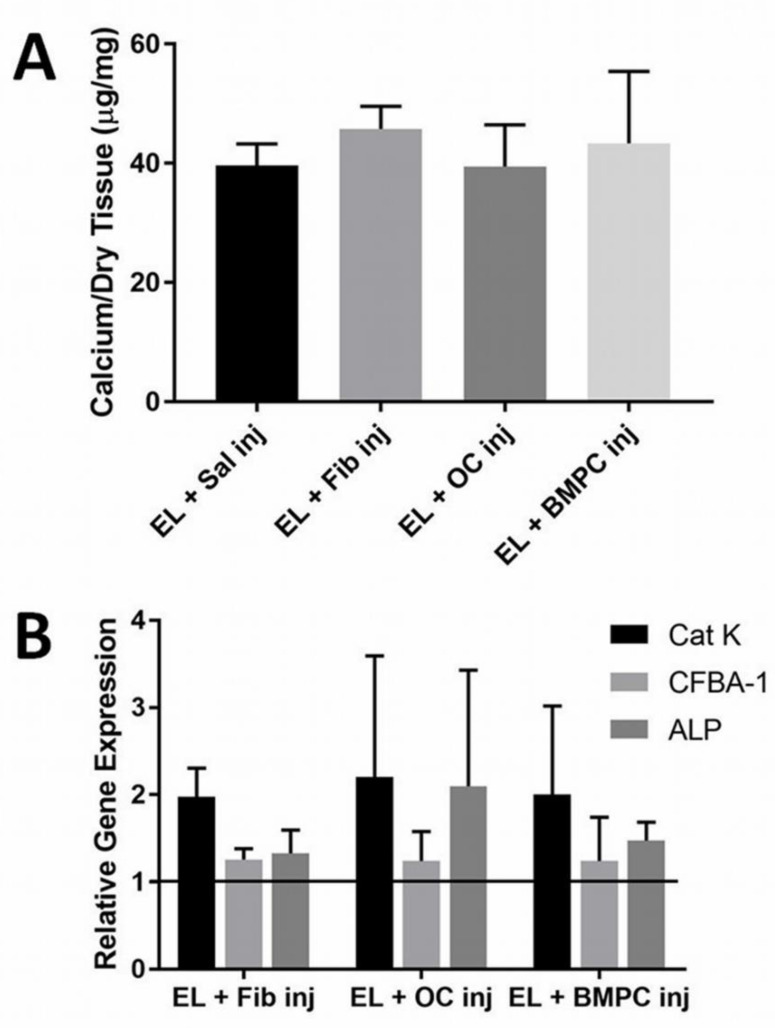
(**A**) The graph depicts the amount of calcium per dry tissue in µg/mg, with elastin and OC injection showing the lowest amount of calcium, though no values were found to be significant. Cells injected at the site of elastin calcification were not effective in reducing calcification (*n* = 4). (**B**) The second image depicts gene expression of each treatment, looking specifically at Cat-K, CFBA-A, and ALP. Gene analysis of the implants shows that cell injections did not reduce bone-specific genes, with all being found at expressions higher than a value of 1 (*n* = 4). Data were analyzed with ANOVA and Student’s *t*-test, with *p* < 0.05.

Seven days after the initial delivery of OCs, elastin was explanted and the amount of calcium deposited was assayed with atomic absorption spectrophotometry. Calcium analysis revealed no significant reduction in the calcification of elastin in the presence of any cell type injected (Fib *p* = 0.67, OC *p* = 0.99, BMPC *p* = 0.89) (Figure 1A). Cellular injections proved ineffective for the reversal of elastin calcification.

This could potentially be due to the fact that the cells did not remain at the implant site long enough to demineralize the calcified elastin. Gene analysis showed that Cathepsin K (Cat-K) levels were two-fold higher in all groups including the fibroblast group compared to the elastin implant control (Figure 1B). Clearly, just delivering cells to the site was enough to increase Cat-K expression given the fact that there was no significant difference in the data (*p* = 0.97). At seven days, early markers of the bone-specific gene, Cbfa-1, remained unchanged while alkaline phosphatase (ALP), which is expressed during the calcification process, was higher in the OC injection group. ALP levels indicate more than just bone formation, also serving as markers of general phosphatase production. OCs use phosphatase in the process of demineralization, potentially pointing to increased ALP gene expression in the OC group.

**Figure 2:**
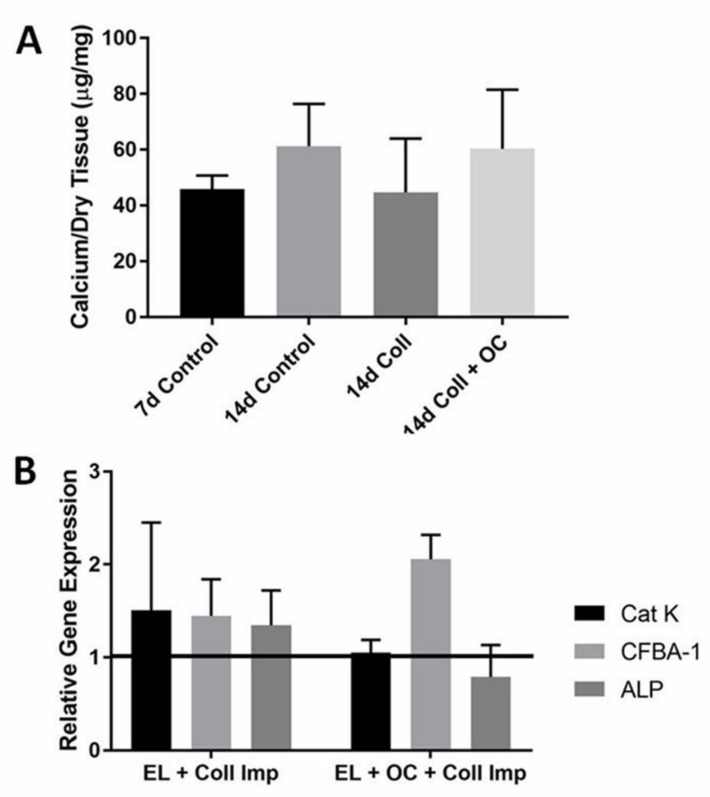
(**A**) The graph depicts calcium per dry tissue in µg/mg for control groups, collagen implant, and collagen implant with OCs included. OCs delivered by collagen gel were ineffective for the reversal of elastin calcification (*n* = 4). (**B**) In the second image, gene analysis results vary but the variance was not enough to affect calcification or show any significant difference, looking specifically at the Cat-K, CFBA-1, and ALP genes. Data were analyzed with ANOVA and Student’s *t*-test, with *p* < 0.05.

Injections of cells suspended in saline were ineffective in reversing elastin calcification, probably due to the difficulty of keeping them in place at the implant site. For this reason, it was determined that some sort of gel delivery system was needed to administer the cells. Thus, collagen gels were used to deliver the cells to the site of calcification. Again, calcium analysis of elastin in the collagen gel treatment showed no reduction in elastin calcification in the presence of OCs (*p* = 0.22) (Figure 2A). Gene analysis showed slightly reduced Cat-K levels in the OC group compared to the day fourteen control, though there is no statistical difference (*p* = 0.38). Cbfa-1 gene was over-expressed in the OC group but ALP levels were reduced (Figure 2B).

The final attempt to reverse elastin calcification using cell therapy was with collagen/alginate beads. In vitro studies were performed first to optimize bead preparation and cell encapsulation. Beads were made with varying ratios of collagen and alginate and the beads averaged 0.2 cm in size. The beads with equal parts collagen and alginate (1:1) were not homogenous in size. The beads made with 7:3 collagen/alginate were weak and mushy. Increasing concentrations of alginate improved the stiffness and handling properties of the beads. The beads with 3:7 collagen/alginate ratio showed better handling properties and were used for further studies.

Cell encapsulation was successful and fluorescently labeled cells were observed within the collagen/alginate beads. The cells remained viable during encapsulation as confirmed by the MTS cell viability assay. While the cells were viable within the bead, picogreen DNA assay showed that they did not migrate from the bead and were attached to the culture dish.

**Figure 3:**
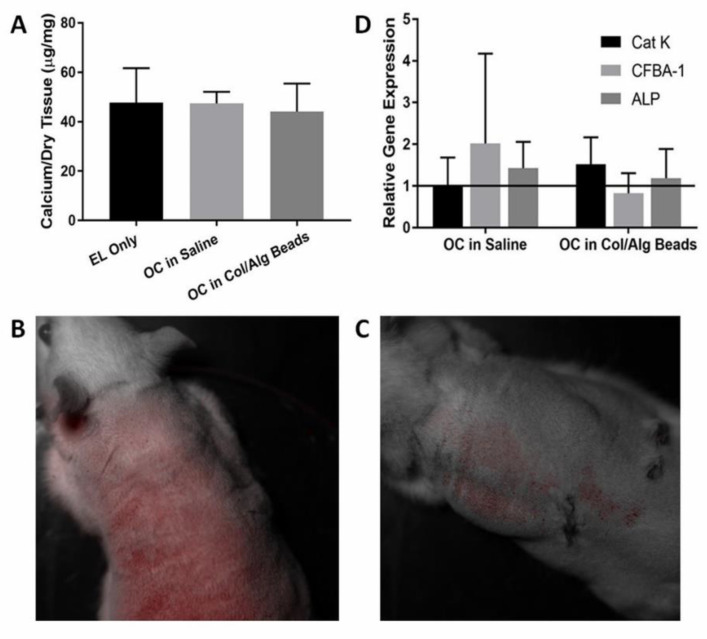
(**A**) The graph shows the calcium per dry tissue (µg/mg) for elastin, OCs, and bead delivery. Delivery of OCs by beads was ineffective in reversing calcification (*n* = 4). (**B**,**C**) Fluorescently labeled OCs seven days after implantation remained localized during and after implantation. In B., the OC saline injection showed cells fluorescing throughout the body of the rat. However, in C., the bead delivery showed cells only fluorescing localized around the injection site. (**D**) The image depicts the relative gene expression of OCs either in saline or encapsulated in beads. Bead delivery showed lower CFBA-1 expression, but no significant difference was observed (*n* = 4). Data were analyzed with ANOVA and Student’s *t*-test, with *p* < 0.05. No statistical difference observed (*p* = 0.96).

Calcium analysis of the implants did not reveal any difference in calcification in the presence of the beads containing OCs (*p* = 0.96) (Figure 3A). However, the collagen/alginate beads kept the cells localized to one specific area (Figure 3B,C). Prior to euthanasia, the animals were imaged using Lumazone imaging technology to visualize the position of the fluorescently labeled cells.

Gene analysis of the implants showed a higher expression of Cat-K, confirming that the collagen/alginate beads kept OCs at the implant site while OCs injected as a suspension in saline were not effective at keeping cells in place (Figure 3D). When OCs were injected in saline bone, related genes were over-expressed. Both Cbfa-1 and ALP levels were nearly two-fold that of the control, but in the group where the OCs were delivered in the beads, the levels remained unchanged as compared to the elastin-only control.

### 3.2. RANKL Injections

**Figure 4:**
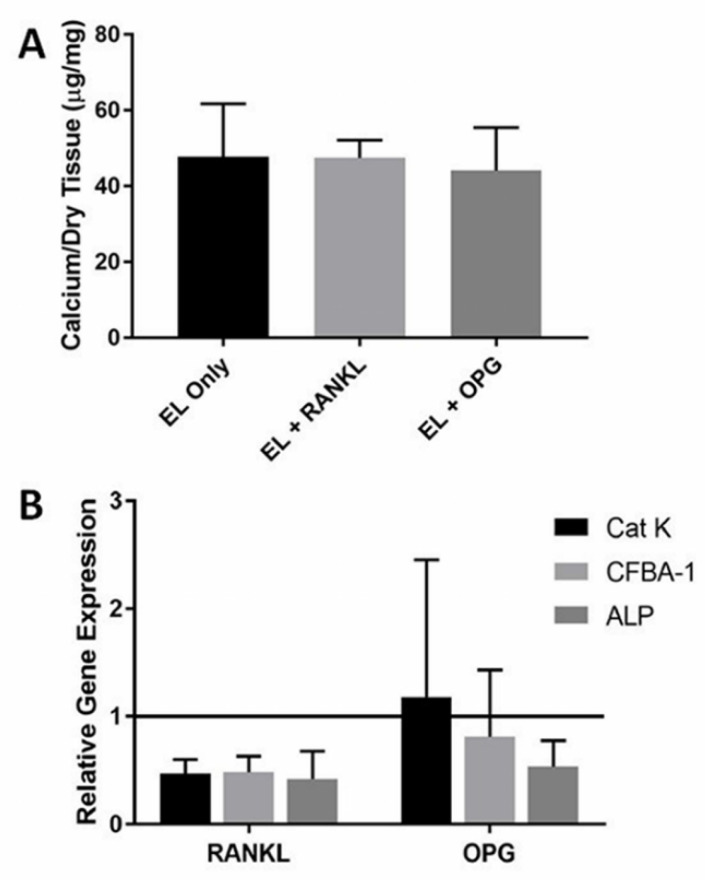
(**A**) RANKL and OPG injections had no effect on elastin calcification, as shown in the calcium per dry tissue weight graph above with no significant differences (*p* = 0.99) (*n* = 4). (**B**) RANKL injections down regulate Cat-K and bone markers CFBA-1 and ALP compared to saline controls while OPG injections only had an effect on ALP (*n* = 4). Data were analyzed with ANOVA and Student’s *t*-test, with *p* < 0.05. No statistical difference was observed.

Calcium analysis of explants showed that RANKL injections were ineffective in reversing elastin calcification. Calcium levels of the RANKL group were not significantly lower than the saline control group, *p* = 0.99 (Figure 4A). OPG injections also had no effect on elastin calcification. Desmosine levels were not decreased due to RANKL or OPG injections, indicating no elastin degradation.

Our group has previously shown that subdermal elastin calcification expresses numerous bone genes and proteins [20]. We wanted to examine if RANKL and OPG injections had any effect on the gene level that may not have been expressed by calcification analysis. We also examined Cat-K gene expression to determine if there was any presence of OCs at the calcification site. RANKL injections decreased the expression of bone proteins Cbfa-1 and ALP but had no effect on reversing calcification. Cat-K levels were also down regulated by RANKL injections, indicating fewer OCs compared to the elastin-only control group (Figure 4B). OPG injections did not exhibit decreased Cat-K levels compared to the control. Though Cbfa-1 gene expression was not affected by OPG injections, ALP was down regulated by OPG injections as compared to control saline injections.

### 3.3. Immediate OC-Encapsulated Gel Application

**Figure 5:**
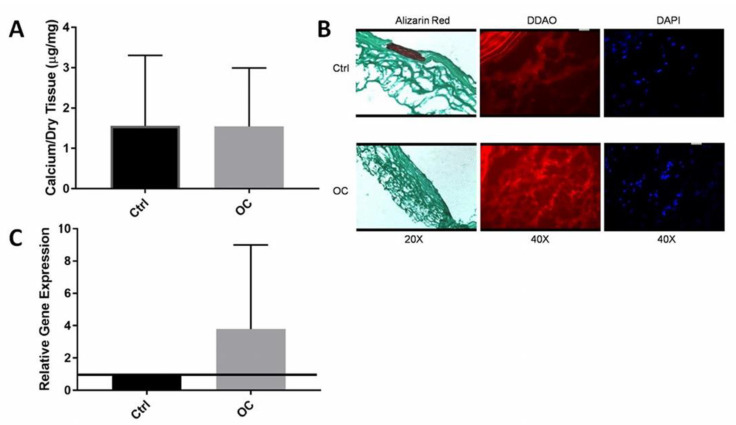
(**A**) Locally delivered OCs did not reduce calcification, as shown in the calcium per dry weight graph, with no difference between controls and OCs (*n* = 5). (**B**) Histological analysis shows the calcification (Alizarin Red) is isolated to the medial layer and although OCs were successfully delivered, they are only in the adventitial layer and did not migrate to the site of calcification (**C**) Gene analysis of Cat-K confirms successful delivery of OCs, with OCs expressing Cat-K at a rate four-fold higher than the control group (*n* = 4). Data were analyzed with ANOVA and Student’s *t*-test, with *p* < 0.05. No statistical differences were observed.

OC presence was also tested by quantitative cathepsin gene activity by RT-PCR (known marker for OCs). Gene analysis confirmed that there was a four-fold increase in Cat-K gene expression in the OC group as compared to controls clearly showing that OCs were present and were expressing Cat-K (Figure 5A).

Frozen tissue sections were stained for calcium by alizarin red. The control group where only collagen gel was used showed strong red staining for calcification in the media of the artery while the OC group showed little to no staining for calcium. (Figure 5B) To test whether OCs that were delivered stayed in place, fluorescent imaging was used. OCs were tagged with DDAO that stains them red. In the OC group, a higher concentration of red staining for cells compared to the control in the adventitia of the aorta was observed. When DAPI counterstain was used for the same section, the red staining corresponded with clumped multinuclear cells. This data suggested that delivered OCs survived and were present at the site at the time of explanation. However, the cells were only seen in the adventitial region of the aorta and not in the media. Calcification was mainly observed in the medial region of the aorta. It seems that OCs did not migrate through to the medial layer where the calcification was located.

Quantitative calcium analysis (Figure 5C) contradicted the histological findings because there was no significant reduction (*p* = 0.77) in calcification by the delivery of OCs. In this study, quantitative calcium data showed far lower calcium levels in both groups compared to our previously published results [21].

Whether OC delivery would increase elastin degradation in the arteries was also investigated because OCs are known to secrete ECM degrading enzymes. Desmosine analysis confirmed that there was no elastin degradation by the delivery of the OCs.

## 4. Discussion

### 4.1. Microbead Osteoclast Delivery

It has been very well documented that vascular calcification is very similar to that of bone mineralization [22,23,24]. The accelerated calcification model used for this study has been previously used by our research group to investigate the fundamental mechanisms of elastin calcification [25]. Our group has also shown that the elastin calcification observed in this model shares many similarities with bone formation, including the presence of osteoblastic cells that are positive for Cbfa-1 surrounding the calcified elastin [26]. The goal was to use OCs, bone-resorbing cells, to reverse elastin calcification [27]. To our knowledge, this was the first attempt to use OCs as cell therapy to reverse elastin calcification.

Cell therapy is a treatment that has been used for numerous other diseases [28]. Recently, cell therapy has proven to be a great treatment in the cardiovascular field. Stem cell use has also shown great potential in treating hearts damaged by myocardial infarction [29]. Transplantation of autologous CD31+ and CD34+ cells induced neovascularization and improved ventricular function after myocardial infarction in pig and rat models [29]. Endothelial progenitor cells isolated from the bone marrow have also been used to achieve cell-based neovascularization in damaged myocardium [29]. The recent findings in the literature using bone marrow-derived stem cells to treat cardiovascular diseases support our hypothesis to use bone marrow-derived OCs to treat vascular elastin-specific calcification.

The results from attempting to reverse elastin calcification through cell therapy using OCs did not align with previous predictions. When cells were suspended in saline, the cells did not remain at the site of calcification and were ineffective at reversing calcification. Rather than being injected in suspension, cells were administered by incorporating OCs into a collagen gel and delivering gel to the site of calcification. Previously we have shown collagen gels to be a great delivery vehicle for OCs [13]. However, the collagen gel did not keep the cells localized long enough for the cells to have an effect on calcification. Finally, collagen/alginate beads were used as a delivery vehicle for the last attempt. It was shown that encapsulated cells in the beads remained viable.

The beads were very effective at keeping the cells localized to a specific area, but the cells did not proliferate and migrate out from the beads to reverse elastin calcification. The collagen/alginate beads were used because of alginate’s ability to degrade slower than collagen alone to ensure the cells would be kept in place and remain at the implant site longer to reverse calcification. The collagen/alginate beads did degrade slower than the collagen gel alone though too slowly. The beads could still be found at the implant site at the time of euthanasia. Studies illustrated the use of alginate for long-term cell entrapment and immobilization as opposed to a short-term delivery vehicle followed by in vivo degradation [30]. The bone protein data were intriguing. When OCs were delivered in saline, their presence caused bone protein levels to also be higher. It is possible that the body is trying to regulate mineral formation and resorption to a certain degree. In bone, OCs regulate the behavior of osteoblasts and vice versa. Thus, it is possible that the presence of OCs allowed increased bone cell type phenotype from nearby cells. When OCs were encapsulated in beads, they remained entrapped and did not cause this response in the nearby cells.

Overall, it is evident that it is difficult to tilt the balance toward osteoclastogenesis from osteoblastogenesis when the calcification has already progressed to a certain degree. It is also very difficult to deliver large cells like OCs to the site of calcification and keep them functional, as attempts to modify delivery methods failed to result in any success. This study demonstrated the need to focus on the optimization of the degradation of the delivery vehicle before attempting to use it for cell therapy to reverse calcification. It is also possible that one can stop the initiation of calcification of elastin as it is a regulated process, but the progression of calcification may occur by physical mineral deposition (without regulation from cells) and the delivery of cells may not prove successful.

In order to further explore the potential of OCs for therapeutic use, a study was designed in which OCs could be recruited rather than delivered, to remove the need for an optimized delivery vehicle. In this way, rather than having to develop individual and specific delivery mechanisms for specific applications, the cells could be recruited through natural functions.

### 4.2. RANKL Injections

Previous approaches of local delivery of OCs were unsuccessful so the focus shifted toward recruitment of native cells by RANKL delivery. It was hypothesized that delivery of small protein molecules like RANKL would be easier than delivering mature OCs.

In bone, homeostasis is maintained through the RANK/RANKL/OPG system [31,32,33,34,35,36]. RANKL (previously called OPGL) was first discovered as a replacement for stromal cells and vitamin D_3_ in OC cell cultures in 1998 by Lacey and associates [36,37,38]. RANKL binds to RANK, which is expressed on pre-osteoclasts. The binding is essential for all aspects of OC function such as differentiation, maturation, fusion, survival, and activity. OPG is a soluble factor produced by some cells that strongly inhibits OC formation in vitro and in vivo. OPG prevents the binding of RANKL to its receptor RANK [33].

Our findings showed that both RANKL and OPG had no effect on reversing elastin calcification that had already progressed to a moderate level. Many factors may be responsible for this ineffectiveness. Protein analysis by ELISA also showed that RANKL levels were not maintained at an active enough level to affect calcification. Thus, injections of RANKL daily at the site were not enough to keep local RANKL levels high enough to cause cell recruitment. It is possible that RANKL was delivered in the solution and was cleared from the area within a few minutes to hours by the circulatory system. More sustained release through delivery systems such as osmotic pumps may have been more effective. Gene analysis for bone markers exhibited down regulation (Cbfa-1 and ALP) in the RANKL group, clearly showing some effect on osteogenesis; however, it was not enough to have an effect on the progression of calcification. It is possible that once moderate calcification takes place, the continuous deposition of the mineral occurs due to physical factors and not by cellular pathways. Injections of OPG did significantly reduce levels of RANKL as confirmed by ELISA clearly showing that OPG delivery did affect RANKL. However, Cat-K (for OCs) and Cbfa-1 (for osteoblast) genes were unaffected compared to the control (saline injections). Only the ALP gene was down regulated in OPG groups, but it also did not inhibit the progression of calcification.

Another potential reason the study did not return expected results may be that the function of RANKL may be context-dependent. Because the RANK/RANKL/OPG pathway has been studied predominantly in typical bone environments, the mechanisms may act differently in a different setting. The literature has shown that a discrepancy exists between the behavior of bone cells and bone-like cells in the vasculature [39]. When bone mass is decreased in diseases like osteoporosis, there is often an associated increase in calcification in the arteries. While the mechanisms behind this phenomenon are not completely clear, this may be related to the ineffective results of the study.

For the final study, after attempting to design an optimum delivery vehicle and attempting to recruit native cells for the medial layer, it was decided to use a different model for calcification. In these previous studies, medial calcification was modeled using porcine elastin. Rather than simulating calcification, the goal was to induce calcification in native arteries to keep conditions clinically accurate.

### 4.3. Immediate OC-Encapsulated Gel Application

Medial elastin-specific calcification is observed in many different diseases such as Monckeberg’s sclerosis, aging, diabetes, and end-stage renal failure. Previous work by our research group has shown that the application of CaCl_2_ to the abdominal aorta of a rat results in medial elastin-associated calcification [22]. Gene analysis of this previous work showed that this model expressed osteogenic genes. Given the similarities between vascular calcification and bone, reversal of medial calcification was attempted with a site-specific delivery of OCs. Native arteries do not express any resemblance to bone, but bone matrix proteins are expressed during diseased states such as Monckeberg’s sclerosis [40].

Attempting to reverse vascular calcification using cell therapy is a novel concept. OCs were successfully delivered to the injured tissue as confirmed by histology and gene expression, but the cells only migrated to the adventitial layer of the artery, which was also observed by histological analysis. Successful delivery of the OCs was not effective at demineralizing the medial vascular calcification because calcium analysis did not show any reduction of calcification in the presence of OCs. The calcification was also isolated to the medial layer of the aorta as confirmed by Alizarin Red staining and the OCs were only in the adventitial layer and did not penetrate to the medial layer to reverse calcification. It is also important to note that these studies did not induce calcification to the level previously shown by our group. Previous studies were able to induce calcification using CaCl_2_ to ~15 ug Ca/mg dry tissue, while this study only calcified to 1.5 ug Ca/mg dry tissue. The reason for this lower calcification is unknown. As no significant calcification was observed in the control group, it may not have been possible to show lower calcification in the OC group. Only one of the control group vessels had a significant calcified area (by alizarin red) while none of the OC group demonstrated any alizarin staining. This warrants speculation that the study was successful but due to insufficient numbers of animals no conclusions could be drawn about whether calcification was reduced or not. This warrants repetition of the study.

## 5. Conclusions

The basis of this study was the similarity between bone mineralization and vascular calcification. It is hypothesized that a site-specific delivery of OCs, the only bone-resorbing cells, would be able to limit and reverse vascular elastin calcification. In previous studies, this was determined to be the most effective method of media supplementation for OC differentiation from BMPCs. These cells were effective at forming resorption pits on hydroxyapatite discs and demineralizing calcified elastin. Next, OCs were cultured to maturity using the chosen method of Vitamin D_3_ with retinoic acid and were also able to form resorption pits on hydroxyapatite discs and demineralize calcified elastin. In vivo studies then confirmed that OCs were able to limit the progression of elastin calcification using a rat subdermal model for accelerated elastin calcification.

Using the same model, the reversal of elastin calcification was attempted. Elastin was allowed to calcify before the cell therapy treatment was applied. Three different delivery methods were used for this attempt at cell therapy. First, cells were suspended in saline and injected directly into the elastin implant. This approach had no effect on reducing calcification due to the cells not remaining at the implant site. Next, cells were encapsulated into a collagen gel for delivery to the implant site. Collagen did not serve as the ideal delivery vehicle because it degraded too quickly and did not keep the cells at the implant site. Lastly, collagen/alginate beads were used as the delivery vehicle for OCs. The beads did keep the cells localized but did not degrade enough during the in vivo study for the OCs to have an effect on calcification.

One potential problem with this therapeutic approach could be that while regulated OCs can resorb bone, introducing them synthetically to the system may not provide enough control or regulation for the resorption process to begin. These OCs could potentially be used as cell therapy, due to the success of in vitro experiments, but a better delivery and cell regulation method is necessary. Using this reasoning, RANKL was used to potentially recruit native OCs to the site of calcification. However, injections of RANKL did not effectively recruit native OCs in vivo to reduce calcification. Though previous research has shown that RANKL inhibition prevents the resorption of bone during osteoporosis in skeletal systems, the in vivo smooth muscle cell model did not reflect that mechanism. According to Chinetti-Gbaguidi, RANKL appears to induce the opposite effect in the calcified vasculature. It is hypothesized that macrophages near these mineral deposits are defective and are unable to differentiate into bone-resorbing OCs [41]. This may be why the in vivo treatment with RANKL was not able to inhibit or reverse the calcification existing in the model.

The second animal model used was a more clinically relevant circulatory model. Creation of medial vascular calcification was attempted and OCs were delivered to the injured tissue to reverse calcification. OCs were successfully delivered to the vascular tissue but only to the adventitial layer and not the medial layer where the calcification was located. The aortic tissue did not calcify to extent of that observed by previous studies.

All institutional and national guidelines for the care and use of laboratory animals were followed and approved by the appropriate institutional committees. No human studies were carried out by the authors for this article.

## Data Availability

The graphed data (gene expression, calcium content, and qualitative imaging) used to support the findings of this study are included within the article.

## Sample Availability

Samples of the compounds are available from the authors.
